# Multiple Introductions and Predominance of Rotavirus Group A Genotype G3P[8] in Kilifi, Coastal Kenya, 4 Years after Nationwide Vaccine Introduction

**DOI:** 10.3390/pathogens9120981

**Published:** 2020-11-24

**Authors:** Mike J. Mwanga, Jennifer R. Verani, Richard Omore, Jacqueline E. Tate, Umesh D. Parashar, Nickson Murunga, Elijah Gicheru, Robert F. Breiman, D. James Nokes, Charles N. Agoti

**Affiliations:** 1 Kenya Medical Research Institute (KEMRI)-Wellcome Trust Research Programme, off Hospital Road, Kilifi 80108, Kenya; mikemwanga6@gmail.com (M.J.M.); nmurunga@kemri-wellcome.org (N.M.); egicheru@kemri-wellcome.org (E.G.); jnokes@kemri-wellcome.org (D.J.N.); 2 Centers for Disease Control and Prevention (CDC), KEMRI Complex, off Mbagathi Way, Village Market, Nairobi 00621, Kenya; qzr7@cdc.gov; 3 Centers for Disease Control and Prevention (CDC), Atlanta, GA 30333, USA; jqt8@cdc.gov (J.E.T.); uap2@cdc.gov (U.D.P.); 4 KEMRI, Center for Global Health Research (KEMRI-CGHR), Kisumu 00202, Kenya; omorerichard@gmail.com; 5 Hubert Department of Global Health, Rollins School of Public Health, Emory University, Atlanta, GA 30322, USA; rfbreiman@emory.edu; 6 School of Life Sciences and Zeeman Institute (SBIDER), The University of Warwick, Coventry CV4 7AL, UK; 7 School of Health and Human Sciences, Pwani University, Kilifi 80108, Kenya

**Keywords:** gastroenteritis, rotavirus, G3[P8], phylogenetics, equine-like

## Abstract

Globally, rotavirus group A (RVA) remains a major cause of severe childhood diarrhea, despite the use of vaccines in more than 100 countries. RVA sequencing for local outbreaks facilitates investigation into strain composition, origins, spread, and vaccine failure. In 2018, we collected 248 stool samples from children aged less than 13 years admitted with diarrheal illness to Kilifi County Hospital, coastal Kenya. Antigen screening detected RVA in 55 samples (22.2%). Of these, VP7 (G) and VP4 (P) segments were successfully sequenced in 48 (87.3%) and phylogenetic analysis based on the VP7 sequences identified seven genetic clusters with six different GP combinations: G3P[8], G1P[8], G2P[4], G2P[8], G9P[8] and G12P[8]. The G3P[8] strains predominated the season (*n* = 37, 67.2%) and comprised three distinct G3 genetic clusters that fell within Lineage I and IX (the latter also known as equine-like G3 Lineage). Both the two G3 lineages have been recently detected in several countries. Our study is the first to document African children infected with G3 Lineage IX. These data highlight the global nature of RVA transmission and the importance of increasing global rotavirus vaccine coverage.

## 1. Introduction

Following progressive introduction of rotavirus vaccines into national immunization programs (NIP) of more than 100 countries since 2006, a significant decline of rotavirus group A (RVA) disease burden has occurred [1,2]. However, despite these successes, RVA remains a leading cause of diarrhea morbidity and mortality [3,4], resulting in an estimated 128,500 deaths annually among under-5-year-olds, a majority occurring in low-income settings [5]. Consistently, licensed oral RVA vaccines have underperformed in low-income settings compared with high-income settings [6,7]. After monovalent Rotarix^®^ vaccine was introduced into Kenya’s NIP in July 2014, with doses given at 6 and 10 weeks of life, a multi-site case-control study found an overall 2-dose vaccine effectiveness of only 64% (95% confidence interval (CI): 35–80%) in under-5-year-olds [8]. In England, the same vaccine showed effectiveness of 77% (95% CI: 66–85%) [9].

In humans, RVA immunity is partly conferred by neutralizing antibodies directed against the VP4 (protease-sensitive) and VP7 (glycoprotein) viral capsid surface proteins that define P and G types, respectively [10]. These two viral proteins are highly diverse, with up to 36 different G and 51 different P types recorded to-date [11], some of which predominantly infect non-human animal species [12]. Among other factors, the higher number of co-circulating GP genotypes in low-income settings has been proposed to be a potential contributor to rotavirus vaccine underperformance [6].

Currently, there are four licensed and WHO pre-qualified RVA vaccines; all live attenuated and administered orally, but with different strain compositions. These are monovalent Rotarix^®^ (G1P[8]), pentavalent RotaTeq^®^ (5 reassortant viruses; G1, G2, G3, G4 and G6 genotypes in combination with P[8]), monovalent ROTAVAC^®^ (G9P[11]) and pentavalent ROTASIIL^®^ (5 reassortant viruses; G1, G2, G3, G4 and G9). All four vaccines were shown to be largely cross-protective against heterotypic strains in both clinical trials and following vaccine implementation in several settings [6,13]. Paradoxically, post-vaccine rollout, outbreaks caused by strains heterotypic to the vaccine in use have been sometimes reported in countries, occurring in patterns seeming to be influenced by the vaccine regimen in use [14,15,16]. 

Recent genotyping studies of RVA have found increased proportions of G2P[4], G3P[8] and G12P[8] genotypes in rotavirus vaccinating countries [14,16,17,18]. These genotypes appeared to play only a minor role in the pre-vaccine era; thus, their increasing prevalence is consistent with increased capacity in escaping vaccine immunity [12,19]. Furthermore, there have been several reports of human infection with equine-like G3 viruses suggestive of greater human vulnerability to antigenically novel RVA strains [20,21,22,23,24,25,26,27,28,29]. At the Kenya Medical Research Institute (KEMRI)—Wellcome Trust Research Programme (KWTRP), we have maintained a RVA surveillance at Kilifi County Hospital (KCH), located in rural coastal Kenya since 2009 [30]. The aim of the current analysis was to determine the genetic relatedness of the strains that were in circulation in the 2018 RVA season in Kilifi, their origins, global phylogenetic context, and role in the local sub-optimal vaccine performance.

## 2. Results

### 2.1. Study Population Characteristics

**Figure 1:**
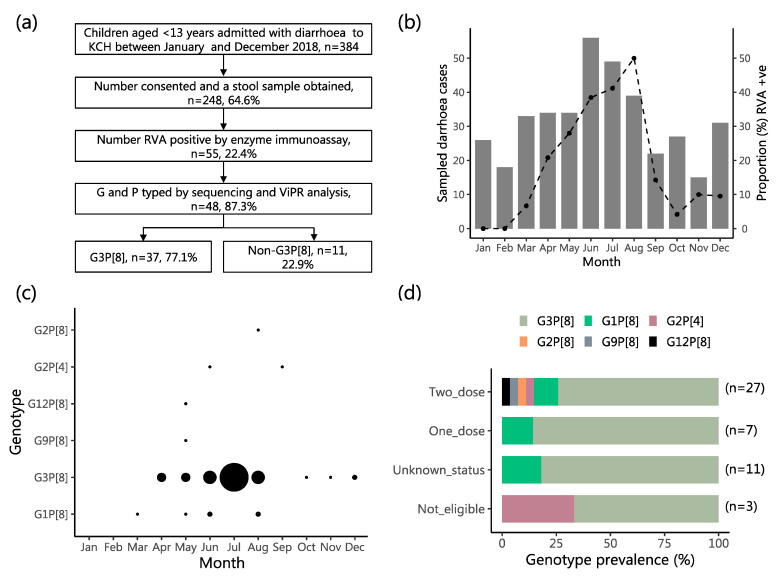
Summary of rotavirus group A (RVA) surveillance in Kilifi County Hospital (KCH) in 2018 and identified genotypes. Panel (**a**) sample flowgram from patient recruitment to VP4 and VP7 genotyping results for the RVA positives. Panel (**b**) monthly cases of diarrhea in children aged less than 13 years recorded at KCH in 2018 (grey bars) compared with monthly proportions of RVA positive samples (black dashed line on the secondary axis). Panel (**c**) the number of RVA positive samples by month in 2018 and by the GP genotype. The black circle size is proportional to the number of samples (the smallest indicates one sample and the largest is 13 samples). Panel (**d**) genotypes identified in children according to rotavirus vaccination status.

Between January and December 2018, 384 children aged less than 13 years were admitted to KCH with diarrhea as one of their illness symptoms. Of these, 208 (54.2%) were Kilifi Health and Demographic surveillance system (KHDSS) area residents (Figure S1). A stool sample was obtained from 248 (64.6%). The main reasons for non-sampling were death (*n* = 13), discharge or transfer before sample collection (*n* = 22), consent refusal (*n* = 52), or other (*n* = 16). Among study eligible children (*n* = 384), the distribution of the sampled and not sampled children differed significantly across age strata (*p* = 0.002) and discharge outcome (*p* < 0.001), Table 1. The distribution of the sampled and not sampled children were similar across sexes and by rotavirus vaccine eligibility status. The majority of the eligible participants were aged less than 2 years (68.2%) and were age eligible to have received one or two doses of rotavirus vaccine (83.6%). By EIA testing, RVA was detected in 55 children (22.2%), Figure 1a, 32 (58.1%) of which were KHDSS area residents. Fifty-one (92.7%) of the RVA positive children were age eligible to have received two doses of the RVA vaccine. Of these, the vaccination status was known for 36 (70.6%), of which 29 (80.6%) were confirmed to have received two doses of Rotarix^®^ vaccine while the remainder (19.4%) received one dose, Table 1. 

### 2.2. Characteristics of the RVA Infections and the Infected Children

RVA prevalence was higher in female compared to male children admitted with diarrhea (29.8% vs. 15.7%, *p* = 0.008), Table 1. RVA was detected in all months of 2018 except January and February Figure 1b. Diarrhea cases peaked in June while RVA prevalence peaked in August (50% of all collected samples were RVA positive). Sequencing and GP typing was successful for 48 (87.3%) of the 55 RVA-positive samples. Five G types (G1, G2, G3, G9 and G12) and two P types (P[4] and P[8]) were identified in the successfully sequenced samples. From these, six GP combinations were identified, namely: G3P[8] (*n* = 37, 77.1%), G1P[8] (*n* = 6, 12.5%), G2P[4] (*n* = 2, 4.2%), G2P[8] (*n* = 1, 2.1%), G9P[8] (*n* = 1, 2.1%) and G12P[8] (*n* = 1, 2.1%). The G3P[8] and G1P[8] strains were the only genotypes detected for > 2 months while the other four genotypes were detected sporadically (1–2 months), Figure 1c. The distribution of the infecting genotype (summarized as G3P[8] versus non-G3P[8]) did not differ significantly by sex, patient age, vaccination status or discharge outcome, Table 2 and Figure 1d. 

### 2.3. Genetic Diversity in the Sequenced Viruses

**Figure 2:**
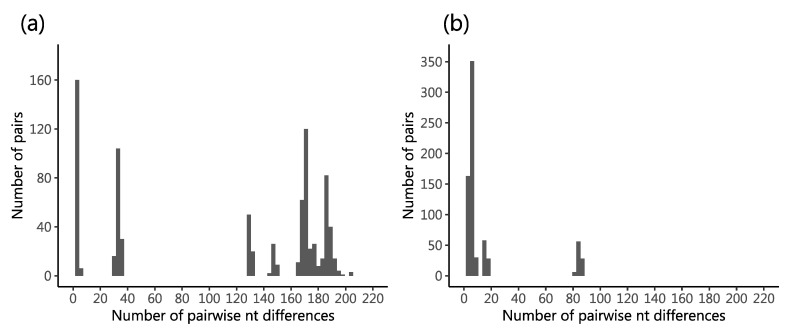
Genetic diversity in the sequenced RVA positives from Kilifi County Hospital (KCH). Panel (**a**) shows the distribution of pairwise nt differences in the sequenced portion of VP7 (644 nt long) of 48 RVA positives. Panel (**b**) shows the distribution of pairwise nt differences in the sequenced portion of VP4 (579 nt long) of 47 RVA positives.

For the VP4 segment, a 579 nt long region (~25%) was recovered for 47 viruses (88.5%) while for the VP7 segment, a 644 nt long region (~65%) was recovered for 48 viruses (87.3%). One virus (KEN/KLF0879/2018), genotyped G9P[8], yielded a significantly shorter VP4 fragment relative to the other viruses (<500 nt) due to low quality sequencing data and was excluded from subsequent analyses. Consistent with the greater number of assigned G types (*n* = 5) compared to P types (*n* = 2) types, the range of pairwise nt differences was much greater in the VP7 (up to 203 nt differences) compared to VP4 segment (up to 87 nt differences), Figure 2a,b, respectively. A multi-modal distribution of nt differences was observed for both VP4 and VP7 segments. A total of 328 (~51%) and 141 (~24%) SNP positions were identified in the sequenced VP7 and VP4 fragments, respectively. Of the 48 sequenced samples, 22 (45.8%) yielded unique VP7 sequences while 17 (36.2%) gave unique VP4 sequences.

### 2.4. Molecular Genetic Clusters

**Figure 3:**
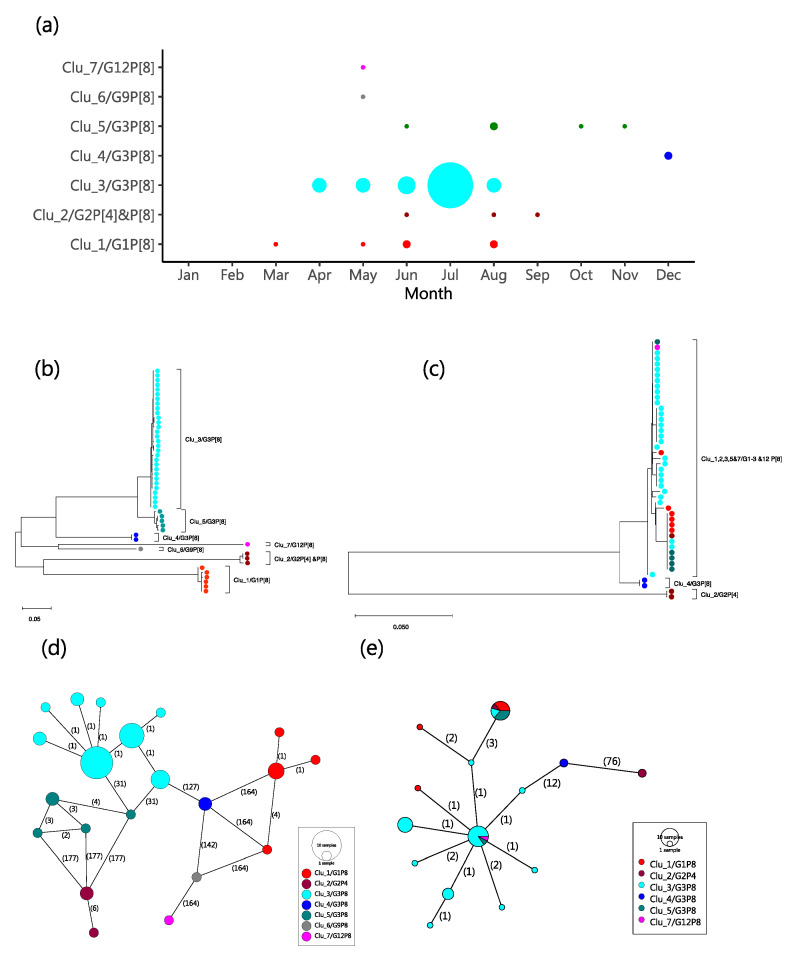
Temporal and genetic relatedness of the sequenced Kilifi rotaviruses. Panel (**a**) number of RVA positive samples by molecular genetic cluster and month. The circle sizes are proportional to the number of samples (the smallest indicates one sample and the largest is 13 samples). Panel (**b**) shows a Maximum Likelihood (ML) tree of the Kilifi 48 VP7 sequences. Panel (**c**) shows an ML tree of the Kilifi 47 VP4 sequences. Panel (**d**) shows the reconstructed POPART minimum spanning network from the 48 VP7 sequences. The vertexes represent the sequenced VP7 haplotypes. The size of the vertex is proportional to the number of haplotypes (identical sequences) and is colored by the assigned molecular genetic cluster. The numbers shown on the edges represent the number of nucleotide changes from one vertex (haplotype) to the next. Panel (**e**) same as panel (**d**) above but for the Kilifi 47 VP4 sequences.

Using the range of pairwise nt differences observed in first modal distribution for the VP7 (0 to 20 nt differences, i.e., >97% nt similarity) to define a molecular genetic cluster, seven G clusters were assigned (named Clu_1-7). Members of a cluster were universally of same G type. All G type sequences identified to be of the same type formed a single cluster except G3P[8] that occurred in three clusters, named Clu_3/G3P[8], Clu_4/G3P[8] and Clu_5/G3P[8]. The temporal pattern of the assigned clusters is shown in Figure 3a. Most of the high incidence months (April to August) had multiple genetic clusters co-circulating, except for July, which had a single G3P[8] cluster. The reconstructed phylogenetic relationship between strains of the different G and P types sequenced is shown in Figure 3b,c. The VP7 phylogeny showed segregation of the seven clusters we identified from the pairwise nt difference analysis. The VP4 phylogeny showed less clear-cut phylogenetic clustering with respect to the assigned genetic clusters. The two phylogenies were not entirely congruent, a feature suggestive of reassortment in the local strains. The minimum spanning networks reconstructed for both the VP7 and VP4 sequences are shown in Figure 3d,e. Viruses in the same genetic cluster consistently had four or less nt differences to the closest next virus within the same genetic cluster. 

### 2.5. Spatial Distribution of the Kilifi G3 Genetic Clusters

A few viruses in different VP7-based genetic clusters had identical VP4 sequences and we explored if these were spatially clustered. Twenty-eight of the 48 genotyped samples were from KHDSS area residents. The geographical distribution of all diarrhea admissions and the RVA positives by genetic cluster is shown in Figure S1. Cases of the predominant Clu_3/G3P[8] strains came from only a few locations although it appeared that road access (especially the Malindi-Mombasa highway) may have played a role in influencing which patients were turning up at KCH due to easier access.

### 2.6. Global Genetic Context of the Kilifi 2018 G3 Strains

**Figure 4:**
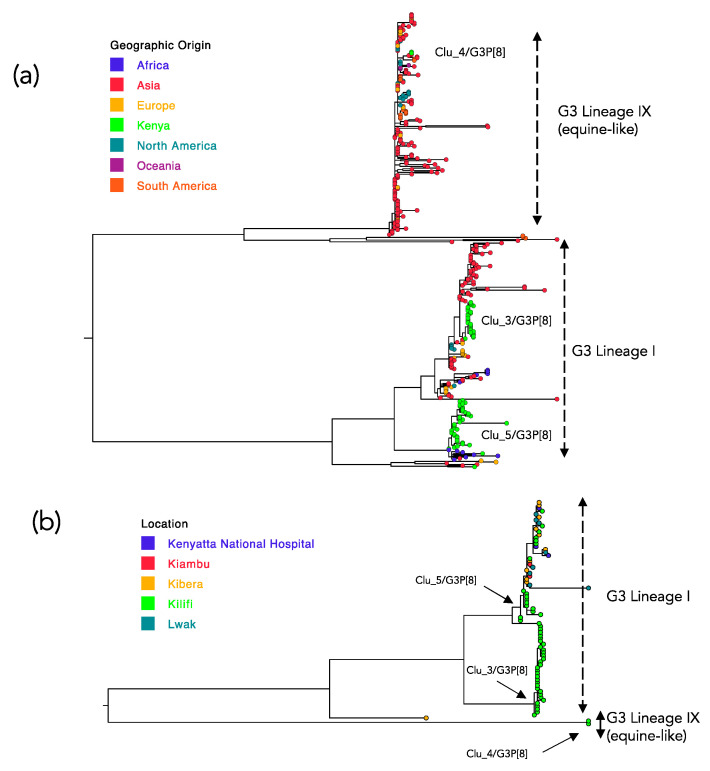
Global phylogeny derived from nucleotide sequences of G3 strains sampled between 2012–2018. (**a**) The phylogenetic tree reconstructed from 375 VP7 sequences of G3 type (338 collated from GenBank sampled across 26 countries including 39 from Kenya, and 37 G3 viruses sequenced in the current study) to determine the lineage and global context of the Kilifi sequences. The countries included were Australia, Belarus, Brazil, China, Dominican Republic, Ethiopia, Hungary, India, Indonesia, Italy, Japan, Kenya, South Korea, Kuwait, Nigeria, Pakistan, Peru, Russia, Spain, Taiwan, Thailand, USA, Uganda and Vietnam. The taxa for Kenya G3 sequences are provided by filled circles colored green and with the assigned Kilifi clusters names indicated next to the branches containing these sequences. Panel (**b**) a phylogeny of all Kenya G3 sequences (*n* = 76). The different colors of the filled circle symbols indicate the Kenya taxa distinguished by their location of sampling. The names assigned to the Kilifi clusters are indicated next to the nodes leading to their branches as similarly shown in panel (**a**).

A total of 338 G3 sequences from 26 countries fully met the criteria for inclusion as comparison data, including 39 previously collected in Kenya. The phylogeny derived from the combined Kilifi and global G3 viruses is shown in Figure 4a while Figure 4b shows the phylogenetic relatedness of all previous G3 sequences of RVA sampled in Kenya (5 locations including Kilifi).

A majority of the global viruses fell within two of nine previously identified G3 lineages [25]; Lineage I and equine-like G3 lineage (named Lineage IX). The Kilifi G3 sequences had representation in both these two lineages: Lineage I (*n* = 35, 94.6%) and equine-like G3 Lineage (*n* = 2, 5.6%). Viruses of the genetic cluster Clu_4/G3P[8] clustered with the equine-like G3 Lineage while the Kilifi G3 Lineage I viruses separated into two groups that corresponded to the Clu_3/G3P[8] cluster (*n* = 30) and the Clu_5/G3P[8] cluster (*n* = 5).The distribution of the pairwise nt differences in the compiled global G3 sequences dataset, like for the Kilifi G3 viruses, showed a multi-modal distribution (figure not shown). The first major trough was observed at 27 nt differences. 

**Figure 5:**
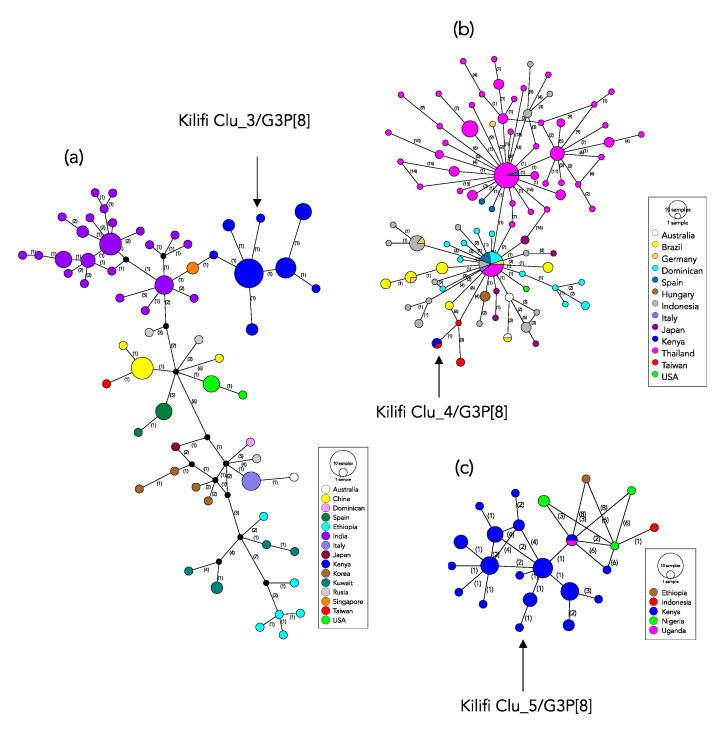
Haplotype network showing relationships of the identified global G3 lineages that included Kilifi viruses. Panel (**a**) shows the network for Lineage I cluster viruses that included the Kilifi Clu_3/G3P[8] strains. The vertices represent the VP7 haplotypes. The size of the vertex is proportional to the number of haplotypes (identical sequences) and is colored by the country of sampling. The numbers shown on the edges represent the number of nucleotide changes from one vertex (haplotype) to the next. Panel (**b**) and (**c**) have the same description as panel (**a**) above but represent Lineage IX (equine-like G3) cluster that included Kilifi Clu_4 G3P[8] and the Lineage I cluster that included Kilifi Clu_5 G3P[8] sequences, respectively.

On applying the threshold used to identify the local molecular genetic clusters (>97% genetic similarity) on the global G3 dataset, 18 clusters were identified (Table S1). Of these, eight were singletons, six comprised of between 2 and 3 members and the remaining four clusters had 10, 47, 116 and 181 members. All the Kilifi G3 viruses fell in the three clusters that had the highest membership overall, Table S1. For each of the three Kilifi G3 genetic clusters we explored their closest genetic relative in the global dataset by network reconstructions (Figure 5). For the Kilifi Clu_3/G3P[8] the closest similar sequences were from India (G3P[8] collected in 2016) and Singapore (G3P[8] collected in 2016) that had 2 nucleotide differences Figure 5a. For the Kilifi Clu_4/G3P[8] (the equine-like G3 Lineage) the closest relative was from Taiwan (G3P[8] collected in 2016) with zero nucleotide difference in the sequenced region Figure 5b. For the Kilifi Clu_5/G3P[8] the closest relatives were from Kenya (G3P[6] collected in 2014) and Uganda (G3P[6] collected in 2013) that had zero and 2 nucleotide difference, respectively, Figure 5c. Overall, within these three major global G3 genetic clusters, clustering by country was common.

## 3. Discussion

Four years after Kenya introduced Rotarix^®^ vaccine into its NIP, multiple RVA GP genotypes circulated during the 2018 season in Kilifi, Kenya, with the G3P[8] genotype predominating at 67.2%. At this study site, the preceding two years (2016 and 2017) were dominated by the G2P[4] and G1P[8] genotypes, respectively, with only six cases of G3P[8] detected from September 2009 to December 2017 [30] and an additional three partially genotyped G3P[x] detected in 2013 [31]. The G3P[8] strains are partially heterotypic to the monovalent Rotarix^®^ vaccine, which is comprised of an attenuated G1P[8] strain. During 2018, this local G3P[8] predominance is consistent with the previously documented season-to-season spatial-temporal fluctuations in the prevalence of RVA genotypes [12], hypothesized to be driven by the prevailing population-level immunity derived from natural infections and the use of vaccines [14]. 

Vaccination records were available for 70.6% of the children with an RVA positive test. Of these, 92.7% were age eligible to have received the two doses of Rotarix^®^ vaccine and, in that subgroup, the vast majority (80.6%) had indeed received the full 2-dose series. However, overall, the vaccination status of these children did not appear to predict either their RVA diagnosis result or the infecting GP genotype. These findings, albeit from a single season and site, suggest that for these children who acquired an RVA infection despite one or two-dose vaccination, host factors rather than viral characteristics or vaccine composition may explain the vaccine failures. A follow-up study is planned. 

At least seven distinct genetic clusters constituted the 2018 coastal Kenya RVA season. The VP7 sequences showed greater genetic diversity and provided a better phylogenetic resolution compared to the VP4 sequences. Each of the identified G types corresponded to a single genetic cluster except G3 viruses that segregated into three genetically distinct clusters. Strikingly, some samples with different G types yielded identical VP4 sequences, indicating that some of the children may have been infected by reassortant viruses or harbored mixed infections [25]. Our analyses improve understanding on the recent composition and transmission patterns of local RVA seasons, providing insight into the design of final stretch RVA control strategies following vaccine introduction. 

Several recent studies have reported the increased proportion of G3P[8] strains, e.g., in Australia [14], Japan [32], Thailand [28], Indonesia [29], Pakistan [33], Dominican Republic [25], Brazil [34], Spain [20], Mozambique [24], Malawi [35] and Botswana [36]. The global G3 sequences available from GenBank showed extensive genetic diversity. The significance of this diversity in relation to human immune recognition should be investigated. Notably, recent years have also observed the emergence and global spread of a new G3 lineage named equine-like G3, of putative equine origin, assigned G3 Lineage IX [25]. Strains of G3 Lineage IX were first detected in 2013 in Japan and have since been widely detected in several other countries (Australia [21], Taiwan (unpublished data in GenBank), Indonesia [29], Thailand [28], USA [26], Dominican Republic [25], Brazil [34], Italy [23], Germany [27], Hungary [22] and Spain [20]). Our study is the first to document African children infection with the G3 Lineage IX. Continued surveillance to monitor whether this particular strain becomes endemic in Kenya and the wider Africa continent in the face of increased RVA vaccine coverage is important to optimize RVA vaccine-mediated control. Notably, recent studies in Botswana [36], Mozambique [24], Malawi [35] and Ethiopia [37] reported increased prevalence of G3 type viruses but sequencing data from these studies are not yet available. 

Based on sequence data deposited in GenBank, the predominant Kilifi G3 cluster (Clu_3/G3P[8]) was the second most common genetic cluster globally. The closest sequences were from Singapore and India, both countries that did not yet have RVA vaccine in their NIP in 2018. The second most prevalent Kilifi G3 genetic cluster was Clu_5/G3P[8]. Notably, this cluster has not been detected frequently around the globe and the closest genetic links were Kenyan strains collected in Kiambu County (Central province) in July and August 2014 [38], Kilifi in 2017, and strains from Ethiopia (collection date: April 2016 [39]) and Uganda (collection date: January 2013 [40]), neighboring countries which included RVA vaccines in their NIP in 2013 and 2018, respectively. Although the Kilifi Clu_4/G3P[8] (equine-like G3 Lineage) was the least prevalent locally, it was the most prevalent globally. The closest relatives to the Kenyan strains were from Taiwan, a country yet to introduce RVA vaccination.

This study had some limitations. First, the sequence data from the cohort represents a single site and one season. Second, we only sequenced portions of the VP4 and VP7 segments. Whereas these data were adequate to assign genotypes, lineages and estimate the number of genetic clusters, whole genome sequences provide a better resolution in examining reassortment events, evolution in internal genes and studying genetic clusters [18,25,41]. Third, to determine the origin and pathways of spread of the imported genetic clusters, background sequence data from more countries and including populations neighboring coastal Kenya would have been ideal. Unfortunately, sequence data in public sequence databases to facilitate such phylogeographic analysis are currently limited. Fourth, the absence of significant epidemiological data for some variables e.g., vaccine status for ~30% of the RVA positive children and geographic origin for children from outside the KHDSS area limited our analyses.

In conclusion, the finding that >20% of diarrheal stools from children admitted to KCH with diarrhea in 2018 were RVA positive highlights that RVA is still a significant contributor to severe childhood diarrhea in coastal Kenya, despite the introduction of Rotarix^®^ into Kenya’s NIP in 2014. The cross-continent detection of the emerging equine-like G3 viruses and other typical human G3 strains demonstrates the global nature of RVA transmission. Strikingly, strains found circulating in the Kilifi population were most closely related to strains circulating in countries that were yet to introduce RVA vaccines into their NIP. This observation reminds of the global connectedness regarding pathogen movement and emphasizes the importance of vaccinating all eligible populations across the world, as failure to do so builds a reservoir for strains that continue to seed transmission in vaccinated populations. Identifying factors responsible for RVA vaccine underperformance in low-income settings is a priority research area that may support efforts to further reduce RVA burden. Our study did not ascertain that viral genetic diversity is a contributor to the vaccine underperformance in this setting. Studies investigating the relationship between RVA vaccine immunogenicity and infant characteristics, such as malnutrition, age at first RVA dose, concomitant receipt of oral polio vaccine (OPV), enteric co-infections and enteric dysbiosis may provide better insight into RVA vaccine performance characteristics. 

## 4. Materials and Methods 

### 4.1. Study Population and Location

KCH is the main referral hospital in Kilifi County (population size ~1.5 million people). The major economic activities in the county are subsistence farming, fishing and tourism [42]. An area around KCH (~900 km^2^ with a population of ~300,000 people) is monitored by the KWTRP and is known as the KHDSS area [42], Figure S1. A high proportion of the patients seeking care at the KCH are KHDSS area residents [42]. Vaccination data of admitted children were collected using an electronic registry [8,43,44].

In the current analysis, stool samples were collected from eligible and consented pediatric patients admitted to KCH between January and December 2018 (the surveillance period), as part of the ongoing rotavirus surveillance program [8,31,43]. All children aged <13 years old admitted with diarrhea (defined as passing three or more watery stools in the last 24-h) were eligible for inclusion [8,31,43]. Following a review of demographic and clinical data collected by a clinical staff, parents or caregivers of eligible children were approached for consent, and a single stool sample was collected. The samples were immediately transferred into a cool box with ice blocks before transportation to the KWTRP for RVA testing and long-term storage at −80 °C. 

### 4.2. Specimen Laboratory Processing

RVA in the stool samples was detected using ProSpecT™ enzyme immunoassay (EIA) kit (Oxoid, Basingstoke, UK) following the manufacturer’s instructions. RVA positive samples were amplified in the VP4 and VP7 segments using One-step Reverse Transcriptase PCR Kit (Qiagen, Valencia, CA, USA) using previously published primers [45,46]. Successful amplification of the target regions was confirmed by the presence of expected bands (VP4: 660 bp and VP7: 881 bp) following gel electrophoresis of the PCR products. Products from successful PCRs were purified using GFX DNA purification kit (GFX-Amersham, Amersham, UK) and sequenced bi-directionally (both in forward and reverse directions) using Big Dye Terminator 3.1 (Applied Biosystems, Foster City, CA, USA) chemistry. The primers used during PCR amplification were used for sequencing on an ABI Prism 3130xl Genetic Analyzer (Applied Biosystems, Foster City, CA, USA).

### 4.3. Genotyping and Phylogenetic Analysis

The sequence reads were assembled using Sequencher v5.4.6 (Gene Codes Corp Inc., Ann Arbor, MI, USA). Nucleotide (nt) sequence alignments were prepared using MAFFT v7.222 and visualized using Aliview v1.8. G and P genotypes were determined using Virus Pathogen Resource (ViPR) online classification tool [47]. The best nt substitution model for the alignments were determined IQ-Tree v1.6.6 [48]. Phylogenetic trees were reconstructed using the maximum likelihood (ML) method in RaxML v8.2.12 [49] and MEGA v7 [50]. Support for the tree branching patterns was evaluated by 1000 bootstrap iterations. 

### 4.4. Genetic Clusters

Molecular genetic clusters were defined from the distribution of pairwise nt differences of VP7 segment sequences. Pairwise nt differences were determined using pairsnp (https://github.com/gtonkinhill/pairsnp/). Viruses within the same molecular genetic clusters were those which pairwise nt differences occurred within the first modal distribution. Using this threshold, clusters were identified using the USEARCH algorithm [51]. Single nucleotide polymorphic (SNP) positions in alignments were assessed using parseSNP [52]. The minimum spanning networks between the RVA positive patients were reconstructed using POPART v1.70 program [53]. 

### 4.5. Comparison Dataset

The phylogenetic context of the locally predominant genotype in global RVA populations was investigated by co-analysis with similar G type strains sequence data deposited in GenBank. The search in GenBank was conducted in October 2020. The criteria for comparison data inclusion were (i) detection in a human stool/rectal swab specimen, (ii) sequence fully overlapping with the VP7 region sequenced for the Kilifi viruses, (iii) information on country and date of sampling available and (iv) sample collected in 2012–2018. G3 sequences collected previously from around Kenya including Kilifi were included in the analysis.

### 4.6. Statistical Analysis

Numerical data were analyzed in STATA v15.1. Continuous variables were summarized using various measures of dispersion. Differences between groups were assessed using a t-test or Wilcoxon rank-sum test. Binary data were summarized using proportions and comparison between groups made using either *χ*^2^ or Fisher’s exact test (depending on group sample size). The 95% CI were presented for proportions and standard deviation for means. A *p*-value of <0.05 was considered significant. 

### 4.7. Data Availability

Partial sequences for the VP7 and VP4 segments reported in this work have been deposited to GenBank database under the sequence accession numbers MN194408-MN194485 for VP7 and MN194325-MN194364 for VP4.

### 4.8. Ethical Statement

Before sample collection informed written consent was obtained from the child’s parent or guardian. The Scientific Ethics Review Unit (SERU) board that sits at KEMRI, Nairobi, approved the study protocols (SERU#3049).

## Figures and Tables

**Table 1:** A comparison of demographic characteristics of children with diarrhea admitted to Kilifi County Hospital (KCH) that were sampled versus those who were not sampled in 2018 and those that were RVA positive versus those that were RVA negative.

Characteristic	All (%)	Sampled (%)	Unsampled (%)	*p* Value ^$^	RVA + ve (%)	RVA − ve (%)	*p* Value *
Number of patients	384	248 (64.6)	136 (35.4)		55 (22.2)	193 (77.8)	
Sex				0.728			0.008
Male	210 (54.7)	134 (54.0)	76 (55.9)		21 (38.2)	113 (58.6)	
Female	174 (45.3)	114 (46.0)	60 (44.1)		34 (61.8)	80 (41.5)	
Age							
Mean (SD ^¶^)	27.4 (29.9)	26.4 (31.8)	29.3 (26.1)	0.352	19.6 (15.0)	28.3 (35.0)	0.073
Median (IQR ^δ^)	16.8 (9.8–29.3)	15.1(9.4–24.1)	19.9 (12.0–39.0)	0.025	15.4 (9.9–20.8)	15.1(8.9–24.9)	1.000
Age group				0.002			0.254
0–11 months	126 (32.8)	92 (37.1)	34 (25.0)		19 (34.6)	73 (37.8)	
12–23 months	136 (35.4)	92 (37.1)	44 (32.4)		25 (45.5)	67 (34.7)	
24–59 months	73 (19.0)	34 (13.7)	39 (28.7)		8 (14.6)	26 (14.0)	
>60 months	49 (12.8)	30 (12.1)	19 (14.0)		3 (5.5)	27 (14.0)	
RVA vaccine eligibility				0.327			0.063
Age eligible 2 dose	317 (82.6)	204 (82.3)	113 (83.1)		51 (92.7)	153 (79.3)	
Age eligible 1 dose	4 (1.0)	4 (1.6)	0 (0.0)		0 (0.0)	4 (2.1)	
Age ineligible	63 (16.4)	40 (16.1)	23 (16.9)		4 (7.3)	36 (18.7)	
Vaccination status (*n* = 321)				0.273			0.209
Two dose eligible & received 2 doses	165 (51.4)	111 (53.4)	54 (47.8)		29 (56.9)	82 (52.2)	
Two dose eligible & received 1 dose	24 (7.5)	17 (8.2)	7 (6.2)		7 (13.7)	10 (6.2)	
One or 2 dose eligible but received none	6 (1.8)	2 (1.0)	4 (3.5)		0 (0.0)	2 (1.3)	
One or 2 dose eligible but status unknown	126 (39.3)	78 (37.5)	48 (42.5)		15 (29.4)	63 (40.1)	
Outcome (*n* = 379)				<0.001			0.194
Died	38 (10.0)	13 (5.3)	25 (18.9)			12 (6.3)	
Alive	341 (90.0)	234 (94.7)	133 (81.1)			180 (93.8)	

^¶^ SD stands for standard deviation; ^δ^ IQR stands for interquartile range; **^$^** *p* value for comparison of sampled and not sampled groups; * *p* value for comparison of RVA positive and negative groups.

**Table 2:** Characteristics of children whom were infected with rotavirus G3P[8] versus those whom were infected with non-G3P[8].

Characteristic	Genotyped RVA (%)	G3P[8] (%)	Non-G3P[8] (%)	*p* Value
Number of patients	48	37 (77.1)	11 (22.9)	
Sex				0.248
Male	19 (39.6)	13 (35.1)	6 (55.6)	
Female	29 (60.4)	24 (64.9)	5 (45.5)	
Age				
Mean (SD ^#^)	19.3 (14.8)	19.2 (13.3)	19.5 (18.6)	0.946
Median (IQR ^δ^)	15.7 (9.9–20.4)	15.9 (9.8–20.4)	15.4 (7.8–23.1)	1.000
Age group				0.770
0–11 months	17 (35.4)	13 (35.1)	4 (36.4)	
12–23 months	22 (45.8)	17 (46.0)	5 (45.6)	
24–59 months	7 (14.6)	6 (16.2)	1 (9.1)	
>60 months	2 (4.2)	1 (2.7)	1 (9.1)	
RVA vaccine eligibility				0.658
Age eligible 2 dose	45 (93.8)	35 (94.6)	10 (90.9)	
Age eligible 1 dose	0 (0.0)	0 (0.0)	0 (0.0)	
Age ineligible	3 (6.3)	2 (5.4)	1 (9.1)	
RVA vaccination status among eligible (*n* = 45)				0.751
Two dose eligible & received two doses	27 (60.0)	20 (57.1)	7 (70.0)	
Two dose eligible & received one dose	7 (15.6)	6 (17.1)	1 (10.0)	
One or 2 dose eligible but received none	0 (0.0)	0 (0.0)	0 (0.0)	
One or 2 dose eligible but status unknown	11 (24.4)	9 (25.7)	2 (20.0)	
Outcome				0.064
Died	1 (2.1)	0 (0.0)	1 (9.1)	
Alive	47 (97.2)	37 (100.0)	10 (90.9)	

^#^ SD stands for standard deviation, ^δ^ IQR stands for interquartile range.

## Supplementary Materials

The following are available online at https://www.mdpi.com/2076-0817/9/12/981/s1, Figure S1: Geographic origin distribution of sampled children who presented with diarrhea symptoms at KCH and were Kilifi Health Demographic Surveillance System (KHDSS) area residents; Table S1: The global distribution of the identified G3 global genetic clusters.

## References

[1] Burnett E., Jonesteller C.L., Tate J.E., Yen C., Parashar U.D. (2017). Global Impact of Rotavirus Vaccination on Childhood Hospitalizations and Mortality From Diarrhea. J. Infect. Dis..

[2] Steele A.D., Groome M.J. (2020). Measuring Rotavirus Vaccine Impact in Sub-Saharan Africa. Clin. Infect. Dis..

[3] Operario D.J., Platts-Mills J.A., Nadan S., Page N., Seheri M., Mphahlele J., Praharaj I., Kang G., Araujo I.T., Leite J.P.G. (2017). Etiology of Severe Acute Watery Diarrhea in Children in the Global Rotavirus Surveillance Network Using Quantitative Polymerase Chain Reaction. J. Infect. Dis..

[4] Iturriza-Gómara M., Jere K.C., Hungerford D., Bar-Zeev N., Shioda K., Kanjerwa O., Houpt E.R., Operario D.J., Wachepa R., Pollock L. (2019). Etiology of Diarrhea Among Hospitalized Children in Blantyre, Malawi, Following Rotavirus Vaccine Introduction: A Case-Control Study. J. Infect. Dis..

[5] Troeger C., Khalil I.A., Rao P.C., Cao S., Blacker B.F., Ahmed T., Armah G., Bines J.E., Brewer T.G., Colombara D.V. (2018). Rotavirus Vaccination and the Global Burden of Rotavirus Diarrhea Among Children Younger Than 5 Years. JAMA Pediatr..

[6] Steele D., Victor J., Carey M., Tate J., Atherly D., Pecenka C., Diaz Z., Parashar U., Kirkwood C. (2019). Experiences with rotavirus vaccines: Can we improve rotavirus vaccine impact in developing countries?. Hum. Vaccines Immunother..

[7] Willame C., Noordegraaf-Schouten M.V., Gvozdenović E., Kochems K., Oordt-Speets A., Praet N., Van Hoorn R., Rosillon D. (2018). Effectiveness of the Oral Human Attenuated Rotavirus Vaccine: A Systematic Review and Meta-analysis—2006–2016. Open Forum Infect. Dis..

[8] Khagayi S., Omore R., Otieno G.P., Ogwel B., Ochieng J.B., Juma J., Apondi E., Bigogo G., Onyango C., Ngama M. (2019). Effectiveness of Monovalent Rotavirus Vaccine Against Hospitalization With Acute Rotavirus Gastroenteritis in Kenyan Children. Clin. Infect. Dis..

[9] Walker J.L., Andrews N.J., Atchison C.J., Collins S., Allen D.J., Ramsay M.E., Ladhani S.N., Thomas S.L. (2019). Effectiveness of oral rotavirus vaccination in England against rotavirus-confirmed and all-cause acute gastroenteritis. Vaccine X.

[10] Nair N., Feng N., Blum L.K., Sanyal M., Ding S., Jiang B., Sen A., Morton J.M., He X.-S., Robinson W.H. (2017). VP4- and VP7-specific antibodies mediate heterotypic immunity to rotavirus in humans. Sci. Transl. Med..

[11] RCWG Rotavirus Classification Working Group: Newly Assigned Genotypes. https://rega.kuleuven.be/cev/viralmetagenomics/virus-classification/rcwg.

[12] Sadiq A., Bostan N., Yinda K.C., Naseem S., Sattar S. (2018). Rotavirus: Genetics, pathogenesis and vaccine advances. Rev. Med. Virol..

[13] Leshem E., Lopman B., Glass R., Gentsch J., Bányai K., Parashar U., Patel M. (2014). Distribution of rotavirus strains and strain-specific effectiveness of the rotavirus vaccine after its introduction: A systematic review and meta-analysis. Lancet Infect. Dis..

[14] Roczo-Farkas S., Kirkwood C.D., Cowley D., Barnes G.L., Bishop R.F., Bogdanovic-Sakran N., Boniface K., Donato C.M., Bines J.E. (2018). The Impact of Rotavirus Vaccines on Genotype Diversity: A Comprehensive Analysis of 2 Decades of Australian Surveillance Data. J. Infect. Dis..

[15] Burke R.M., Tate J.E., Barin N., Bock C., Bowen M.D., Chang D., Gautam R., Han G., Holguin J., Huynh T. (2018). Three Rotavirus Outbreaks in the Postvaccine Era—California, 2017. MMWR Morb. Mortal. Wkly. Rep..

[16] Pitzer V.E., Bilcke J., Heylen E., Crawford F.W., Callens M., De Smet F., Van Ranst M., Zeller M., Matthijnssens J. (2015). Did Large-Scale Vaccination Drive Changes in the Circulating Rotavirus Population in Belgium?. Sci. Rep..

[17] Ianiro G., Micolano R., Di Bartolo I., Scavia G., Monini M., RotaNet-Italy Study Group (2019). Group A rotavirus surveillance before vaccine introduction in Italy, September 2014 to August 2017. Eurosurveillance.

[18] Ogden K.M., Tan Y., Akopov A., Stewart L.S., McHenry R., Fonnesbeck C.J., Piya B., Carter M.H., Fedorova N.B., Halpin R.A. (2018). Multiple Introductions and Antigenic Mismatch with Vaccines May Contribute to Increased Predominance of G12P[8] Rotaviruses in the United States. J. Virol..

[19] Santos N., Hoshino Y. (2005). Global distribution of rotavirus serotypes/genotypes and its implication for the development and implementation of an effective rotavirus vaccine. Rev. Med. Virol..

[20] Arana A., Montes M., Jere K.C., Alkorta M., Iturriza-Gómara M., Cilla G. (2016). Emergence and spread of G3P[8] rotaviruses possessing an equine-like VP7 and a DS-1-like genetic backbone in the Basque Country (North of Spain), 2015. Infect. Genet. Evol..

[21] Cowley D., Donato C.M., Roczo-Farkas S., Kirkwood C.D. (2016). Emergence of a novel equine-like G3P[8] inter-genogroup reassortant rotavirus strain associated with gastroenteritis in Australian children. J. Gen. Virol..

[22] Dóró R., Marton S., Bartókné A.H., Lengyel G., Agócs Z., Jakab F., Bányai K. (2016). Equine-like G3 rotavirus in Hungary, 2015—Is it a novel intergenogroup reassortant pandemic strain?. Acta Microbiol. Immunol. Hung..

[23] Esposito S., Camilloni B., Bianchini S., Ianiro G., Polinori I., Farinelli E., Monini M., Principi N. (2019). First detection of a reassortant G3P[8] rotavirus A strain in Italy: A case report in an 8-year-old child. Virol. J..

[24] João E.D., Munlela B., Chissaque A., Chilaúle J., Langa J., Augusto O., Boene S.S., Anapakala E., Sambo J., Guimarães E. (2020). Molecular Epidemiology of Rotavirus A Strains Pre- and Post-Vaccine (Rotarix^®^) Introduction in Mozambique, 2012–2019: Emergence of Genotypes G3P[4] and G3P[8]. Pathogens.

[25] Katz E.M., Esona M.D., Betrapally N., Leon L.A.D.L.C.D., Neira Y.R., Rey G.J., Bowen M.D. (2019). Whole-gene analysis of inter-genogroup reassortant rotaviruses from the Dominican Republic: Emergence of equine-like G3 strains and evidence of their reassortment with locally-circulating strains. Virology.

[26] Perkins C., Mijatovic-Rustempasic S., Ward M.L., Cortese M.M., Bowen M.D. (2017). Genomic Characterization of the First Equine-Like G3P[8] Rotavirus Strain Detected in the United States. Genome Announc..

[27] Pietsch C., Liebert U. (2018). Molecular characterization of different equine-like G3 rotavirus strains from Germany. Infect. Genet. Evol..

[28] Tacharoenmuang R., Komoto S., Guntapong R., Upachai S., Singchai P., Ide T., Fukuda S., Ruchusatsawast K., Sriwantana B., Tatsumi M. (2020). High prevalence of equine-like G3P[8] rotavirus in children and adults with acute gastroenteritis in Thailand. J. Med. Virol..

[29] Utsumi T., Wahyuni R.M., Doan Y.H., Dinana Z., Soegijanto S., Fujii Y., Juniastuti, Yamani L.N., Matsui C., Deng L. (2018). Equine-like G3 rotavirus strains as predominant strains among children in Indonesia in 2015-2016. Infect. Genet. Evol..

[30] Mwanga M.J., Owor B.E., Ochieng J.B., Ngama M.H., Ogwel B., Onyango C., Juma J., Njeru R., Gicheru E., Otieno G.P. (2020). Rotavirus group A genotype circulation patterns across Kenya before and after nationwide vaccine introduction, 2010–2018. BMC Infect. Dis..

[31] Owor B.E., Mwanga M.J., Njeru R., Mugo R., Ngama M., Otieno G.P., Nokes D.J., Agoti C.N. (2018). Molecular characterization of rotavirus group A strains circulating prior to vaccine introduction in rural coastal Kenya, 2002–2013. Wellcome Open Res..

[32] Thongprachum A., Chan-It W., Khamrin P., Okitsu S., Nishimura S., Kikuta H., Yamamoto A., Sugita K., Baba T., Mizuguchi M. (2013). Reemergence of new variant G3 rotavirus in Japanese pediatric patients, 2009–2011. Infect. Genet. Evol..

[33] Umair M., Abbasi B.H., Sharif S., Alam M.M., Rana M.S., Mujtaba G., Arshad Y., Fatmi M.Q., Zaidi S.Z. (2018). High prevalence of G3 rotavirus in hospitalized children in Rawalpindi, Pakistan during 2014. PLoS ONE.

[34] Guerra S.F.S., Soares L.S., Lobo P.S., Júnior E.T.P., Júnior E.C.S., Bezerra D.A.M., Vaz L.R., Linhares A.C., Mascarenhas J.D.P. (2016). Detection of a novel equine-like G3 rotavirus associated with acute gastroenteritis in Brazil. J. Gen. Virol..

[35] Mhango C., Mandolo J.J., Chinyama E., Wachepa R., Kanjerwa O., Malamba-Banda C., Matambo P.B., Barnes K.G., Chaguza C., Shawa I.T. (2020). Rotavirus Genotypes in Hospitalized Children with Acute Gastroenteritis Before and After Rotavirus Vaccine Introduction in Blantyre, Malawi, 1997–2019. J. Infect. Dis..

[36] Mokomane M., Esona M., Bowen M.D., Tate J., Steenhoff A., Lechiile K., Gaseitsiwe S., Seheri L., Magagula N., Weldegebriel G. (2019). Diversity of Rotavirus Strains Circulating in Botswana before and after introduction of the Monovalent Rotavirus Vaccine. Vaccine.

[37] Abebe A., Getahun M., Mapaseka S.L., Beyene B., Assefa E., Teshome B., Tefera M., Kebede F., Habtamu A., Haile-Mariam T. (2018). Impact of rotavirus vaccine introduction and genotypic characteristics of rotavirus strains in children less than 5 years of age with gastroenteritis in Ethiopia: 2011–2016. Vaccine.

[38] Wandera E.A., Komoto S., Mohammad S., Ide T., Bundi M., Nyangao J., Kathiiko C., Odoyo E., Galata A., Miring’U G. (2019). Genomic characterization of uncommon human G3P[6] rotavirus strains that have emerged in Kenya after rotavirus vaccine introduction, and pre-vaccine human G8P[4] rotavirus strains. Infect. Genet. Evol..

[39] Gelaw A., Pietsch C., Liebert U.G. (2018). Molecular epidemiology of rotaviruses in Northwest Ethiopia after national vaccine introduction. Infect. Genet. Evol..

[40] Bwogi J., Jere K.C., Karamagi C., Byarugaba D.K., Namuwulya P., Baliraine F.N., Desselberger U., Iturriza-Gomara M. (2017). Whole genome analysis of selected human and animal rotaviruses identified in Uganda from 2012 to 2014 reveals complex genome reassortment events between human, bovine, caprine and porcine strains. PLoS ONE.

[41] Jere K.C., Chaguza C., Bar-Zeev N., Lowe J., Peno C., Kumwenda B., Nakagomi O., Tate J.E., Parashar U.D., Heyderman R.S. (2017). Emergence of Double- and Triple-Gene Reassortant G1P[8] Rotaviruses Possessing a DS-1-Like Backbone after Rotavirus Vaccine Introduction in Malawi. J. Virol..

[42] Scott J.A.G., Bauni E., Moisi J.C., Ojal J., Gatakaa H., Nyundo C., Molyneux C.S., Kombe F., Tsofa B., Marsh K. (2012). Profile: The Kilifi Health and Demographic Surveillance System (KHDSS). Int. J. Epidemiol..

[43] Otieno G.P., Bottomley C., Khagayi S., Adetifa I., Ngama M., Omore R., Ogwel B., Owor B.E., Bigogo G., Ochieng J.B. (2019). Impact of the Introduction of Rotavirus Vaccine on Hospital Admissions for Diarrhea Among Children in Kenya: A Controlled Interrupted Time-Series Analysis. Clin. Infect. Dis..

[44] Adetifa I.M., Bwanaali T., Wafula J., Mutuku A., Karia B., Makumi A., Mwatsuma P., Bauni E., Hammitt L.L., Nokes D.J. (2016). Cohort Profile: The Kilifi Vaccine Monitoring Study. Int. J. Epidemiol..

[45] Gómara M.I., Cubitt D., Desselberger U., Gray J. (2001). Amino Acid Substitution within the VP7 Protein of G2 Rotavirus Strains Associated with Failure To Serotype. J. Clin. Microbiol..

[46] Simmonds M.K., Armah G., Asmah R., Banerjee I., Damanka S., Esona M., Gentsch J.R., Gray J.J., Kirkwood C., Page N. (2008). New oligonucleotide primers for P-typing of rotavirus strains: Strategies for typing previously untypeable strains. J. Clin. Virol..

[47] Pickett B.E., Greer D., Zhang Y., Stewart L., Zhou L., Sun G., Gu Z., Kumar S., Zaremba S., Larsen C.N. (2012). Virus Pathogen Database and Analysis Resource (ViPR): A Comprehensive Bioinformatics Database and Analysis Resource for the Coronavirus Research Community. Viruses.

[48] Nguyen L.-T., Schmidt H.A., Von Haeseler A., Minh B.Q. (2015). IQ-TREE: A Fast and Effective Stochastic Algorithm for Estimating Maximum-Likelihood Phylogenies. Mol. Biol. Evol..

[49] Stamatakis A. (2015). Using RAxML to Infer Phylogenies. Curr. Protoc. Bioinform..

[50] Kumar S., Stecher G., Tamura K. (2016). MEGA7: Molecular Evolutionary Genetics Analysis Version 7.0 for Bigger Datasets. Mol. Biol. Evol..

[51] Edgar R.C. (2010). Search and clustering orders of magnitude faster than BLAST. Bioinformatics.

[52] Taylor N.E., Greene E.A. (2003). PARSESNP: A tool for the analysis of nucleotide polymorphisms. Nucleic Acids Res..

[53] Leigh J.W., Bryant D. (2015). POPART: Full-feature software for haplotype network construction. Methods Ecol. Evol..

