# Supplementary material for: Multiple Introductions and Predominance of Rotavirus Group A Genotype G3P[8] in Kilifi, Coastal Kenya, 4 Years after Nationwide Vaccine Introduction
Source: Pathogens. 2020 Nov 24;9(12):981. doi: 10.3390/pathogens9120981 (PMC7761311; doi:10.3390/pathogens9120981)
Supplement: Supplementary file 1 [file pathogens-09-00981-s001.pdf]

pathogens

MDPI

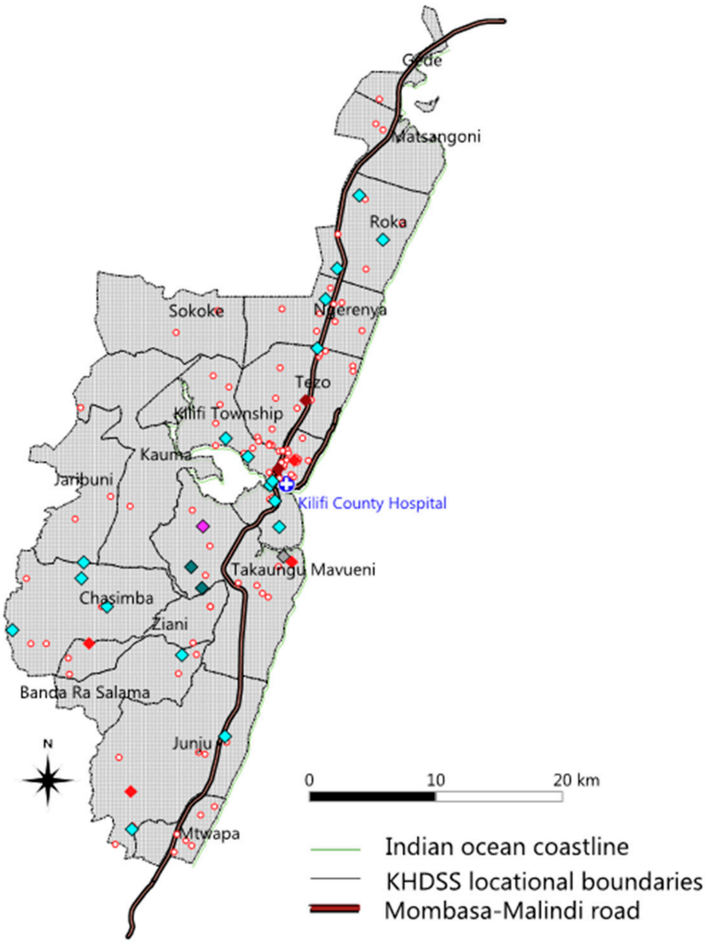

Figure S1. Geographic origin distribution of sampled children who presented with diarrhea symptoms at KCH and were KHDSS residents. The diamonds indicate RVA positives and are colored by genetic cluster (see Figure 3 for details. The red empty circles indicate RVA negative cases.

Table S1: The global distribution of the identified G3 global genetic clusters

Global cluster name	Number of sequences	Includes Kilifi cluster	Years detected	Countries detected
**Global/Clu_1**	**181**	**Clu_4/G3P[8]**	**2013, 2014, 2015, 2016, 2017, 2018**	**Australia, Taiwan, Japan, Indonesia, Thailand, USA, Dominican, Brazil, Italy, Germany, Hungary, Spain, Kenya**
Global/Clu_2	116	Clu_3/G3P[8]	2013, 2014, 2015, 2016, 2018	India, Pakistan, China, Taiwan, Korea, Japan, Singapore, Australia, Italy, Spain, Russia, USA, Dominican, Ethiopia, Kenya
**Global/Clu_3**	**47**	**Clu_5/G3P[8]**	**2013, 2014, 2016, 2017, 2018**	**Ethiopia, Kenya, Nigeria, Uganda, Indonesia**
Global/Clu_4	3	-	2014	Peru
Global/Clu_5	1	-	2014	China
Global/Clu_6	**10**		2015, 2016, 2018	**Kenya, Pakistan, Indonesia**
Global/Clu_7	1	-	2016	Indonesia
**Global/Clu_8**	**2**	**-**	**2013, 2016**	**Kenya, China**
Global/Clu_9	1	-	2016	Kuwait
Global/Clu_10	2	-	2013, 2014	Belarus
**Global/Clu_11**	**2**		**2013, 2015**	**Kenya**
Global/Clu_12	1	-	2013	Nigeria
Global/Clu_13	1	-	2015	Thailand
Global/Clu_14	1	-	2016	Thailand
Global/Clu_15	1	-	2016	Thailand
Global/Clu_16	2	-	2014, 2016	Viet Nam, Korea
Global/Clu_17	1	-	2013	China
Global/Clu_18	2	-	2015, 2016	Indonesia
